# Supplementary material for: Detecting SARS-CoV-2 variants in wastewater and their correlation with circulating variants in the communities
Source: Sci Rep. 2022 Sep 27;12:16141. doi: 10.1038/s41598-022-20219-2 (PMC9514676; doi:10.1038/s41598-022-20219-2)
Supplement: Supplementary file 1 — Supplementary Information. [file 41598_2022_20219_MOESM1_ESM.pdf]

Supplementary Information

Detecting the prevalence of SARS-CoV-2 Variants in the wastewater and their correlation with circulating variants in the communities

Lin Li[1], Timsy Uppal[2], Paul D. Hartley[3], Andrew Gorzalski[4], Mark Pandori[4,5], Michael A. Picker[6], Subhash C. Verma[2,*], Krishna Pagilla[1,*]

Affiliations:

[1] Department of Civil and Environmental Engineering, University of Nevada, MS258, Reno, NV 89557, USA

[2] Department of Microbiology and Immunology, University of Nevada, Reno School of Medicine, MS320, Reno NV, 89557, USA

[3] Nevada Genomics Center, University of Nevada, Reno, NV, 89557, USA

[4] Nevada State Public Health Laboratory, Reno, NV, USA

[5] Department of Pathology and Laboratory Medicine, University of Nevada, Reno School of Medicine, Reno, NV, USA

[6] Southern Nevada Public Health Laboratory of the Southern Nevada Health District, Las Vegas, NV, USA

[*] To whom correspondence should be addressed:

Subhash C. Verma scverma@med.unr.edu and **Krishna Pagilla** pagilla@unr.edu

SARS-CoV-2 recovery efficiency, and quality control

Pepper mild mottle virus (PMMoV) was analyzed as a process control because it is an endogenous wastewater organism and can validate the virus concentration method. PMMoVs were quantified according to PMMoV RT-qPCR Kit for Wastewater (Promega, Madison, WI, USA). For the samples that didn't yield a positive amplification by RT-qPCR but yielded a detectable amplification of PMMoV, we assume the concentration of SARS-CoV-2 is below the LoD in the wastewater. Human coronavirus OC43 strain (OC43) was used as a surrogate to study the virus recovery rate in the concentration method because of the enveloped structure, which is similar with SARS-CoV-2. 100 µL of OC43 strain were spiked into 180 mL wastewater, followed by the Amicon ultrafiltration method. The HCoV-OC43 was quantified according to the method by Uppal et al., (2021). The recovery rate was calculated according to Equation (1).

$$\text{Recovery rate} = (\text{OC43 recovered})/(\text{HCoV\_OC43 spiked}) \cdot 100\% \quad (1)$$

The recovery OC43 in untreated wastewater was 24 ± 2 %, whereas it was 22 ± 10 % in spiked PBS. No significant difference was observed between the two groups (Mann-Whitney test, P value = 0.85). The result is shown in the Supplementary Figure 1.

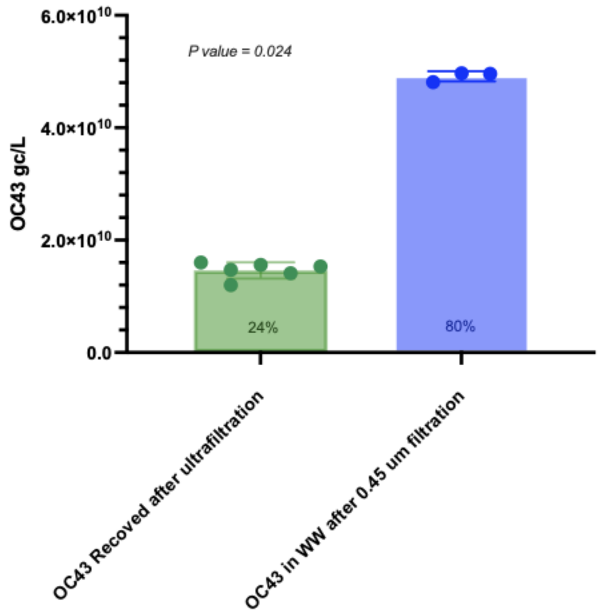

Supplementary Figure 1. Recovery rate of OC43 after Amicon ultrafiltration.

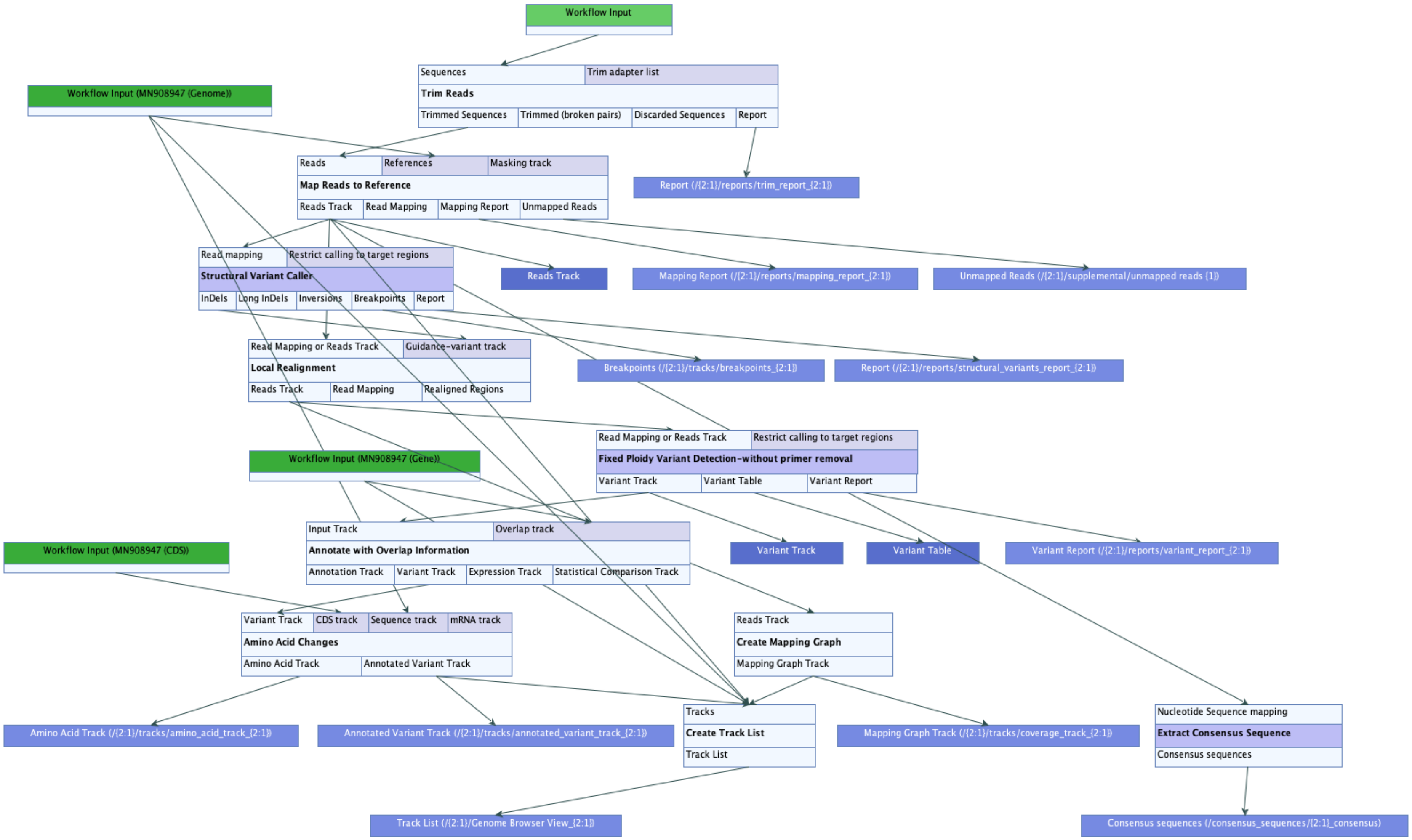

Supplementary Figure 2. Workflow used for the identification of variants of CLC Genomic Workbench v21.

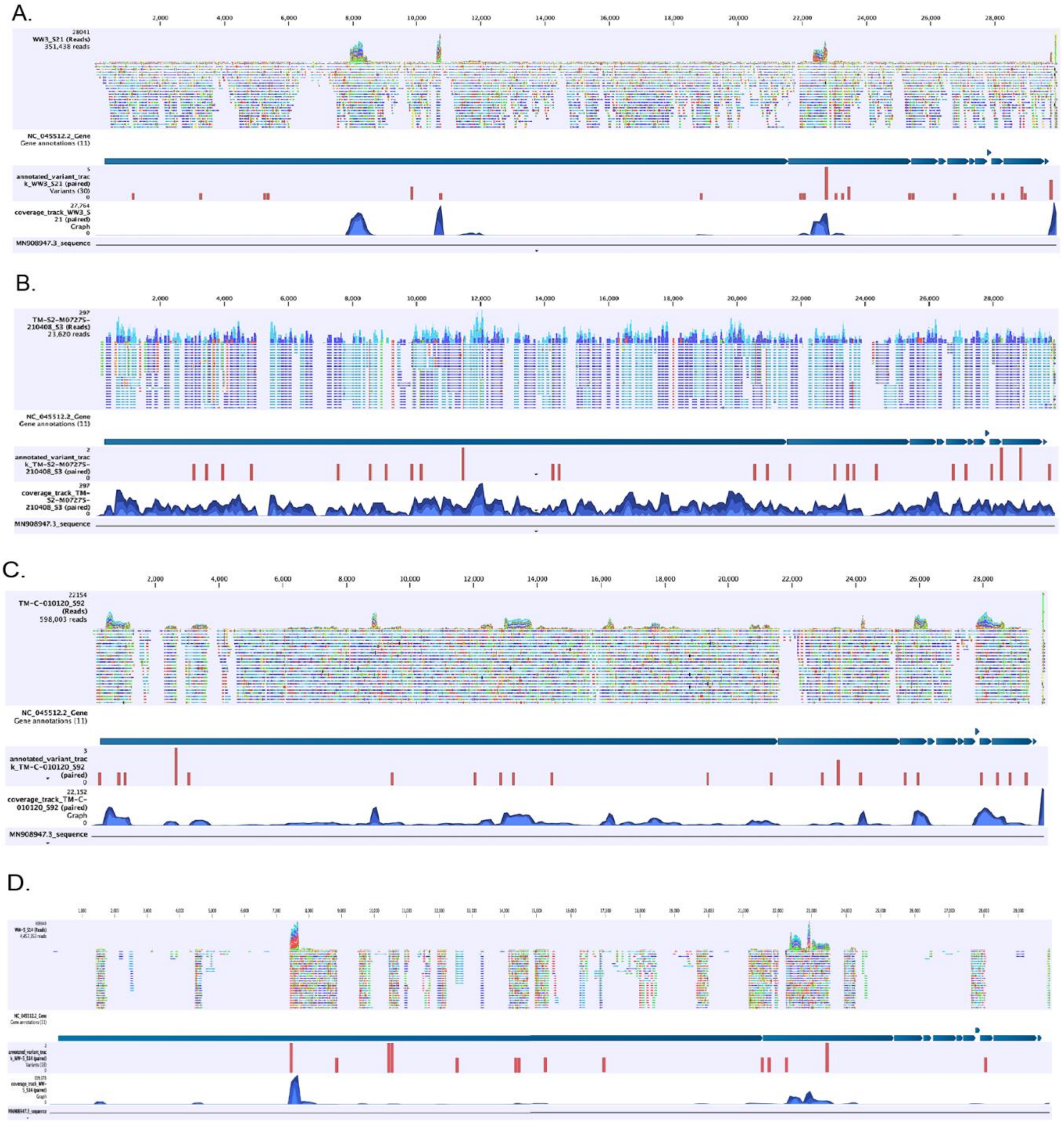

Supplementary Figure 3. Mapping of the reads from wastewater specimens to the reference SARS-CoV-2 (Wuhan-Hu1), accession number NC_045512.2. **A.** Sequence reads in the

wastewater from June, 2021; **B.** Sequence reads in the wastewater from March 2021; C. Sequence reads in the wastewater from January 2021; D. Sequence reads in the wastewater from Novemver 2020.

Table S1: GISAID Accession Numbers

EPI_ISL_1135856	EPI_ISL_1135941	EPI_ISL_1136250	EPI_ISL_1136292
EPI_ISL_1135857	EPI_ISL_1135942	EPI_ISL_1136251	EPI_ISL_1136294
EPI_ISL_1135860	EPI_ISL_1135944	EPI_ISL_1136253	EPI_ISL_1136295
EPI_ISL_1135861	EPI_ISL_1135947	EPI_ISL_1136254	EPI_ISL_1136296
EPI_ISL_1135862	EPI_ISL_1135948	EPI_ISL_1136255	EPI_ISL_1136297
EPI_ISL_1135864	EPI_ISL_1135949	EPI_ISL_1136256	EPI_ISL_1136298
EPI_ISL_1135865	EPI_ISL_1135950	EPI_ISL_1136257	EPI_ISL_1136299
EPI_ISL_1135868	EPI_ISL_1135956	EPI_ISL_1136258	EPI_ISL_1136300
EPI_ISL_1135870	EPI_ISL_1135965	EPI_ISL_1136259	EPI_ISL_1136301
EPI_ISL_1135871	EPI_ISL_1135967	EPI_ISL_1136260	EPI_ISL_1136302
EPI_ISL_1135873	EPI_ISL_1135969	EPI_ISL_1136261	EPI_ISL_1136303
EPI_ISL_1135874	EPI_ISL_1135970	EPI_ISL_1136262	EPI_ISL_1136304
EPI_ISL_1135875	EPI_ISL_1135975	EPI_ISL_1136263	EPI_ISL_1136305
EPI_ISL_1135876	EPI_ISL_1135978	EPI_ISL_1136264	EPI_ISL_1136306
EPI_ISL_1135883	EPI_ISL_1135979	EPI_ISL_1136265	EPI_ISL_1136307
EPI_ISL_1135884	EPI_ISL_1135981	EPI_ISL_1136266	EPI_ISL_1136307
EPI_ISL_1135887	EPI_ISL_1135984	EPI_ISL_1136267	EPI_ISL_1136308
EPI_ISL_1135888	EPI_ISL_1135985	EPI_ISL_1136268	EPI_ISL_1136309
EPI_ISL_1135889	EPI_ISL_1135986	EPI_ISL_1136269	EPI_ISL_1136310
EPI_ISL_1135891	EPI_ISL_1135989	EPI_ISL_1136270	EPI_ISL_1136311
EPI_ISL_1135892	EPI_ISL_1135993	EPI_ISL_1136271	EPI_ISL_1136312
EPI_ISL_1135894	EPI_ISL_1135994	EPI_ISL_1136272	EPI_ISL_1136313
EPI_ISL_1135898	EPI_ISL_1135997	EPI_ISL_1136273	EPI_ISL_1136314
EPI_ISL_1135903	EPI_ISL_1135998	EPI_ISL_1136274	EPI_ISL_1136315
EPI_ISL_1135905	EPI_ISL_1136002	EPI_ISL_1136275	EPI_ISL_1136316
EPI_ISL_1135906	EPI_ISL_1136004	EPI_ISL_1136276	EPI_ISL_1136317
EPI_ISL_1135908	EPI_ISL_1136007	EPI_ISL_1136277	EPI_ISL_1136318
EPI_ISL_1135910	EPI_ISL_1136008	EPI_ISL_1136278	EPI_ISL_1136319
EPI_ISL_1135914	EPI_ISL_1136014	EPI_ISL_1136279	EPI_ISL_1136320
EPI_ISL_1135915	EPI_ISL_1136052	EPI_ISL_1136280	EPI_ISL_1136321
EPI_ISL_1135919	EPI_ISL_1136053	EPI_ISL_1136281	EPI_ISL_1136322
EPI_ISL_1135920	EPI_ISL_1136054	EPI_ISL_1136282	EPI_ISL_1136323
EPI_ISL_1135923	EPI_ISL_1136058	EPI_ISL_1136283	EPI_ISL_1136324
EPI_ISL_1135924	EPI_ISL_1136059	EPI_ISL_1136284	EPI_ISL_1136325
EPI_ISL_1135927	EPI_ISL_1136060	EPI_ISL_1136285	EPI_ISL_1136326
EPI_ISL_1135928	EPI_ISL_1136061	EPI_ISL_1136286	EPI_ISL_1136327
EPI_ISL_1135930	EPI_ISL_1136062	EPI_ISL_1136287	EPI_ISL_1136328
EPI_ISL_1135932	EPI_ISL_1136063	EPI_ISL_1136288	EPI_ISL_1136329
EPI_ISL_1135938	EPI_ISL_1136247	EPI_ISL_1136289	EPI_ISL_1136330
EPI_ISL_1135939	EPI_ISL_1136248	EPI_ISL_1136290	EPI_ISL_1136331
EPI_ISL_1135940	EPI_ISL_1136249	EPI_ISL_1136291	EPI_ISL_1136332

EPI_ISL_1136333	EPI_ISL_1136381	EPI_ISL_1136459	EPI_ISL_1136602
EPI_ISL_1136334	EPI_ISL_1136382	EPI_ISL_1136460	EPI_ISL_1136603
EPI_ISL_1136335	EPI_ISL_1136383	EPI_ISL_1136464	EPI_ISL_1136604
EPI_ISL_1136336	EPI_ISL_1136384	EPI_ISL_1136479	EPI_ISL_1136605
EPI_ISL_1136337	EPI_ISL_1136385	EPI_ISL_1136482	EPI_ISL_1136606
EPI_ISL_1136338	EPI_ISL_1136386	EPI_ISL_1136485	EPI_ISL_1136607
EPI_ISL_1136339	EPI_ISL_1136387	EPI_ISL_1136486	EPI_ISL_1136608
EPI_ISL_1136340	EPI_ISL_1136388	EPI_ISL_1136487	EPI_ISL_1136610
EPI_ISL_1136342	EPI_ISL_1136389	EPI_ISL_1136489	EPI_ISL_1136611
EPI_ISL_1136343	EPI_ISL_1136390	EPI_ISL_1136490	EPI_ISL_1136616
EPI_ISL_1136344	EPI_ISL_1136391	EPI_ISL_1136491	EPI_ISL_1136617
EPI_ISL_1136345	EPI_ISL_1136392	EPI_ISL_1136493	EPI_ISL_1136618
EPI_ISL_1136346	EPI_ISL_1136396	EPI_ISL_1136494	EPI_ISL_1136622
EPI_ISL_1136347	EPI_ISL_1136399	EPI_ISL_1136499	EPI_ISL_1136623
EPI_ISL_1136348	EPI_ISL_1136402	EPI_ISL_1136500	EPI_ISL_1136624
EPI_ISL_1136349	EPI_ISL_1136403	EPI_ISL_1136501	EPI_ISL_1136625
EPI_ISL_1136350	EPI_ISL_1136404	EPI_ISL_1136502	EPI_ISL_1136626
EPI_ISL_1136351	EPI_ISL_1136405	EPI_ISL_1136503	EPI_ISL_1136627
EPI_ISL_1136353	EPI_ISL_1136406	EPI_ISL_1136504	EPI_ISL_1136628
EPI_ISL_1136354	EPI_ISL_1136407	EPI_ISL_1136519	EPI_ISL_1136629
EPI_ISL_1136355	EPI_ISL_1136410	EPI_ISL_1136537	EPI_ISL_1136631
EPI_ISL_1136356	EPI_ISL_1136411	EPI_ISL_1136538	EPI_ISL_1136632
EPI_ISL_1136357	EPI_ISL_1136413	EPI_ISL_1136539	EPI_ISL_1136633
EPI_ISL_1136358	EPI_ISL_1136418	EPI_ISL_1136540	EPI_ISL_1136634
EPI_ISL_1136359	EPI_ISL_1136424	EPI_ISL_1136541	EPI_ISL_1136635
EPI_ISL_1136360	EPI_ISL_1136425	EPI_ISL_1136543	EPI_ISL_1136636
EPI_ISL_1136361	EPI_ISL_1136427	EPI_ISL_1136544	EPI_ISL_1136637
EPI_ISL_1136363	EPI_ISL_1136428	EPI_ISL_1136547	EPI_ISL_1136638
EPI_ISL_1136364	EPI_ISL_1136430	EPI_ISL_1136550	EPI_ISL_1136648
EPI_ISL_1136365	EPI_ISL_1136436	EPI_ISL_1136551	EPI_ISL_1136649
EPI_ISL_1136366	EPI_ISL_1136438	EPI_ISL_1136552	EPI_ISL_1136650
EPI_ISL_1136367	EPI_ISL_1136439	EPI_ISL_1136567	EPI_ISL_1136651
EPI_ISL_1136368	EPI_ISL_1136440	EPI_ISL_1136568	EPI_ISL_1136652
EPI_ISL_1136369	EPI_ISL_1136441	EPI_ISL_1136578	EPI_ISL_1136674
EPI_ISL_1136370	EPI_ISL_1136442	EPI_ISL_1136579	EPI_ISL_1136675
EPI_ISL_1136371	EPI_ISL_1136444	EPI_ISL_1136580	EPI_ISL_1136676
EPI_ISL_1136373	EPI_ISL_1136445	EPI_ISL_1136581	EPI_ISL_1136677
EPI_ISL_1136374	EPI_ISL_1136449	EPI_ISL_1136582	EPI_ISL_1136679
EPI_ISL_1136375	EPI_ISL_1136450	EPI_ISL_1136589	EPI_ISL_1136680
EPI_ISL_1136376	EPI_ISL_1136451	EPI_ISL_1136590	EPI_ISL_1136681
EPI_ISL_1136377	EPI_ISL_1136455	EPI_ISL_1136592	EPI_ISL_1136682
EPI_ISL_1136378	EPI_ISL_1136456	EPI_ISL_1136593	EPI_ISL_1136685
EPI_ISL_1136379	EPI_ISL_1136457	EPI_ISL_1136594	EPI_ISL_1136687
EPI_ISL_1136380	EPI_ISL_1136458	EPI_ISL_1136596	EPI_ISL_1136688

EPI_ISL_1136689	EPI_ISL_1136804	EPI_ISL_1136871	EPI_ISL_1263152
EPI_ISL_1136723	EPI_ISL_1136805	EPI_ISL_1136872	EPI_ISL_1263153
EPI_ISL_1136724	EPI_ISL_1136806	EPI_ISL_1136873	EPI_ISL_1263154
EPI_ISL_1136725	EPI_ISL_1136807	EPI_ISL_1136874	EPI_ISL_1263155
EPI_ISL_1136726	EPI_ISL_1136808	EPI_ISL_1136875	EPI_ISL_1263156
EPI_ISL_1136727	EPI_ISL_1136809	EPI_ISL_1136878	EPI_ISL_1263158
EPI_ISL_1136731	EPI_ISL_1136810	EPI_ISL_1136879	EPI_ISL_1263159
EPI_ISL_1136733	EPI_ISL_1136811	EPI_ISL_1136880	EPI_ISL_1263160
EPI_ISL_1136734	EPI_ISL_1136816	EPI_ISL_1136881	EPI_ISL_1263161
EPI_ISL_1136735	EPI_ISL_1136817	EPI_ISL_1136889	EPI_ISL_1263162
EPI_ISL_1136736	EPI_ISL_1136819	EPI_ISL_1136891	EPI_ISL_1263163
EPI_ISL_1136737	EPI_ISL_1136820	EPI_ISL_1136895	EPI_ISL_1263164
EPI_ISL_1136738	EPI_ISL_1136821	EPI_ISL_1136898	EPI_ISL_1263165
EPI_ISL_1136739	EPI_ISL_1136823	EPI_ISL_1136899	EPI_ISL_1263166
EPI_ISL_1136742	EPI_ISL_1136833	EPI_ISL_1136900	EPI_ISL_1263167
EPI_ISL_1136746	EPI_ISL_1136835	EPI_ISL_1136901	EPI_ISL_1263168
EPI_ISL_1136747	EPI_ISL_1136838	EPI_ISL_1136902	EPI_ISL_1263169
EPI_ISL_1136748	EPI_ISL_1136839	EPI_ISL_1136904	EPI_ISL_1263170
EPI_ISL_1136755	EPI_ISL_1136840	EPI_ISL_1136905	EPI_ISL_1263171
EPI_ISL_1136756	EPI_ISL_1136841	EPI_ISL_1136908	EPI_ISL_1263172
EPI_ISL_1136757	EPI_ISL_1136842	EPI_ISL_1136909	EPI_ISL_1263173
EPI_ISL_1136758	EPI_ISL_1136843	EPI_ISL_1136910	EPI_ISL_1263175
EPI_ISL_1136759	EPI_ISL_1136844	EPI_ISL_1136911	EPI_ISL_1263176
EPI_ISL_1136760	EPI_ISL_1136845	EPI_ISL_1137302	EPI_ISL_1263177
EPI_ISL_1136761	EPI_ISL_1136846	EPI_ISL_1137303	EPI_ISL_1263178
EPI_ISL_1136762	EPI_ISL_1136847	EPI_ISL_1219807	EPI_ISL_1263179
EPI_ISL_1136763	EPI_ISL_1136848	EPI_ISL_1219820	EPI_ISL_1263180
EPI_ISL_1136764	EPI_ISL_1136849	EPI_ISL_1219829	EPI_ISL_1263181
EPI_ISL_1136765	EPI_ISL_1136851	EPI_ISL_1219865	EPI_ISL_1263182
EPI_ISL_1136766	EPI_ISL_1136852	EPI_ISL_1219866	EPI_ISL_1263183
EPI_ISL_1136771	EPI_ISL_1136853	EPI_ISL_1219867	EPI_ISL_1263184
EPI_ISL_1136772	EPI_ISL_1136854	EPI_ISL_1219869	EPI_ISL_1263185
EPI_ISL_1136774	EPI_ISL_1136858	EPI_ISL_1219901	EPI_ISL_1263186
EPI_ISL_1136775	EPI_ISL_1136859	EPI_ISL_1219905	EPI_ISL_1263187
EPI_ISL_1136776	EPI_ISL_1136861	EPI_ISL_1219906	EPI_ISL_1263188
EPI_ISL_1136788	EPI_ISL_1136862	EPI_ISL_1219907	EPI_ISL_1263189
EPI_ISL_1136790	EPI_ISL_1136863	EPI_ISL_1219908	EPI_ISL_1263190
EPI_ISL_1136791	EPI_ISL_1136864	EPI_ISL_1219909	EPI_ISL_1263191
EPI_ISL_1136792	EPI_ISL_1136865	EPI_ISL_1219910	EPI_ISL_1270787
EPI_ISL_1136795	EPI_ISL_1136866	EPI_ISL_1263103	EPI_ISL_1295902
EPI_ISL_1136796	EPI_ISL_1136867	EPI_ISL_1263104	EPI_ISL_1295904
EPI_ISL_1136797	EPI_ISL_1136868	EPI_ISL_1263105	EPI_ISL_1295905
EPI_ISL_1136802	EPI_ISL_1136869	EPI_ISL_1263110	EPI_ISL_1295907
EPI_ISL_1136803	EPI_ISL_1136870	EPI_ISL_1263151	EPI_ISL_1295908

EPI_ISL_1295911
EPI_ISL_1295913
EPI_ISL_1295914
EPI_ISL_1295915
EPI_ISL_1295916
EPI_ISL_1295917
EPI_ISL_1295918
EPI_ISL_1295919
EPI_ISL_1295920
EPI_ISL_1295922
EPI_ISL_1295923
EPI_ISL_1295924
EPI_ISL_1295925
EPI_ISL_1295926
EPI_ISL_1295927
EPI_ISL_1295928
EPI_ISL_1315092
EPI_ISL_1315093
EPI_ISL_1315108
EPI_ISL_1315109
EPI_ISL_1315110
EPI_ISL_1315111
EPI_ISL_1315112
EPI_ISL_1315113
EPI_ISL_1315114
EPI_ISL_1315115
EPI_ISL_1315116
EPI_ISL_1315117
EPI_ISL_1315118
EPI_ISL_1315119
EPI_ISL_1315120
EPI_ISL_1315121
EPI_ISL_1315122
EPI_ISL_1315123
EPI_ISL_1315124
EPI_ISL_1315125
EPI_ISL_1323639
EPI_ISL_1323640
EPI_ISL_1323641
EPI_ISL_1323642
EPI_ISL_1323643
EPI_ISL_1323644
EPI_ISL_1323645
EPI_ISL_1323646
EPI_ISL_1323647
EPI_ISL_1323648
EPI_ISL_1323649
EPI_ISL_1323650
EPI_ISL_1323651
EPI_ISL_1323652
EPI_ISL_1324786
EPI_ISL_1324787
EPI_ISL_1324788
EPI_ISL_1324789
EPI_ISL_1324790
EPI_ISL_1324791
EPI_ISL_1324792
EPI_ISL_1324793
EPI_ISL_1324794
EPI_ISL_1324795
EPI_ISL_1324796
EPI_ISL_1324797
EPI_ISL_1324798
EPI_ISL_1364431
EPI_ISL_1364434
EPI_ISL_1364435
EPI_ISL_1364443
EPI_ISL_1364444
EPI_ISL_1364445
EPI_ISL_1364446
EPI_ISL_1364447
EPI_ISL_1364448
EPI_ISL_1364449
EPI_ISL_1378664
EPI_ISL_1378665
EPI_ISL_1378666
EPI_ISL_1378668
EPI_ISL_1378669
EPI_ISL_1399165
EPI_ISL_1399166
EPI_ISL_1399167
EPI_ISL_1399168
EPI_ISL_1399169
EPI_ISL_1399170
EPI_ISL_1399171
EPI_ISL_1399172
EPI_ISL_1399173
EPI_ISL_1399174
EPI_ISL_1399175
EPI_ISL_1399176
EPI_ISL_1399177
EPI_ISL_1399178
EPI_ISL_1399179
EPI_ISL_1404604
EPI_ISL_1404605
EPI_ISL_1404606
EPI_ISL_1404607
EPI_ISL_1404608
EPI_ISL_1404609
EPI_ISL_1404610
EPI_ISL_1404611
EPI_ISL_1404612
EPI_ISL_1404613
EPI_ISL_1423001
EPI_ISL_1423002
EPI_ISL_1423003
EPI_ISL_1423004
EPI_ISL_1423005
EPI_ISL_1469498
EPI_ISL_1469499
EPI_ISL_1469500
EPI_ISL_1469501
EPI_ISL_1469502
EPI_ISL_1469503
EPI_ISL_1469504
EPI_ISL_1469505
EPI_ISL_1469506
EPI_ISL_1469507
EPI_ISL_1469508
EPI_ISL_1469509
EPI_ISL_1469510
EPI_ISL_1469511
EPI_ISL_1469512
EPI_ISL_1469513
EPI_ISL_1469514
EPI_ISL_1469515
EPI_ISL_1469516
EPI_ISL_1469517
EPI_ISL_1469518
EPI_ISL_1469519
EPI_ISL_1469520
EPI_ISL_1469521
EPI_ISL_1469522
EPI_ISL_1469523
EPI_ISL_1469524
EPI_ISL_1469525
EPI_ISL_1469526
EPI_ISL_1469528
EPI_ISL_1469530
EPI_ISL_1469531
EPI_ISL_1469532
EPI_ISL_1469533
EPI_ISL_1469534
EPI_ISL_1469535
EPI_ISL_1469536
EPI_ISL_1469537
EPI_ISL_1469538
EPI_ISL_1469539
EPI_ISL_1469545
EPI_ISL_1483257
EPI_ISL_1483258
EPI_ISL_1483259
EPI_ISL_1483266
EPI_ISL_1483267
EPI_ISL_1483268
EPI_ISL_1483269
EPI_ISL_1483270
EPI_ISL_1483271
EPI_ISL_1483272
EPI_ISL_1483273
EPI_ISL_1483274
EPI_ISL_1483275
EPI_ISL_1483276
EPI_ISL_1483277
EPI_ISL_1483278
EPI_ISL_1493146
EPI_ISL_1493147
EPI_ISL_1493148
EPI_ISL_1493149
EPI_ISL_1493150
EPI_ISL_1493151
EPI_ISL_1493152
EPI_ISL_1493153
EPI_ISL_1493154
EPI_ISL_1493155
EPI_ISL_1493156

EPI_ISL_1493157
EPI_ISL_1493158
EPI_ISL_1493160
EPI_ISL_1493161
EPI_ISL_1493162
EPI_ISL_1493163
EPI_ISL_1493164
EPI_ISL_1501680
EPI_ISL_1501681
EPI_ISL_1501682
EPI_ISL_1511637
EPI_ISL_1511638
EPI_ISL_1511639
EPI_ISL_1575284
EPI_ISL_1575285
EPI_ISL_1575286
EPI_ISL_1575287
EPI_ISL_1575288
EPI_ISL_1575289
EPI_ISL_1575290
EPI_ISL_1663903
EPI_ISL_1663904
EPI_ISL_1663905
EPI_ISL_1663906
EPI_ISL_1663907
EPI_ISL_1663908
EPI_ISL_1663909
EPI_ISL_1663910
EPI_ISL_1675149
EPI_ISL_1822611
EPI_ISL_1822613
EPI_ISL_1822614
EPI_ISL_1822615
EPI_ISL_1822616
EPI_ISL_1822617
EPI_ISL_1822618
EPI_ISL_1822620
EPI_ISL_1822622
EPI_ISL_1822624
EPI_ISL_1961036
EPI_ISL_1961037
EPI_ISL_1961038
EPI_ISL_1961039
EPI_ISL_1961040
EPI_ISL_1961041
EPI_ISL_1961042
EPI_ISL_1961043
EPI_ISL_1961044
EPI_ISL_1961045
EPI_ISL_2032397
EPI_ISL_2032399
EPI_ISL_2032401
EPI_ISL_2032403
EPI_ISL_2032405
EPI_ISL_2032407
EPI_ISL_2032410
EPI_ISL_2032412
EPI_ISL_2032414
EPI_ISL_2032416
EPI_ISL_2032418
EPI_ISL_2032422
EPI_ISL_2032424
EPI_ISL_2032427
EPI_ISL_2032429
EPI_ISL_2032431
EPI_ISL_2032433
EPI_ISL_2032435
EPI_ISL_2032437
EPI_ISL_2211083
EPI_ISL_2211086
EPI_ISL_2211088
EPI_ISL_2211091
EPI_ISL_2211096
EPI_ISL_2211103
EPI_ISL_2211110
EPI_ISL_2211116
EPI_ISL_2211118
EPI_ISL_2211123
EPI_ISL_2308352
EPI_ISL_2991999
EPI_ISL_2992024
EPI_ISL_2992031
EPI_ISL_2992033
EPI_ISL_2992037
EPI_ISL_2992038
EPI_ISL_2992039
EPI_ISL_2992040
EPI_ISL_2992041
EPI_ISL_3760874
EPI_ISL_4877378
EPI_ISL_4877379
EPI_ISL_4877389
EPI_ISL_4877395
EPI_ISL_4877396
EPI_ISL_4877397
EPI_ISL_4877398
EPI_ISL_4877399
EPI_ISL_4877416
EPI_ISL_4877417
EPI_ISL_4877418
EPI_ISL_4877419
EPI_ISL_4877420
EPI_ISL_4877421
EPI_ISL_4877422
EPI_ISL_4877423
EPI_ISL_4877424
EPI_ISL_4877426
EPI_ISL_4877427
EPI_ISL_4877428
EPI_ISL_4877429
EPI_ISL_4877430
EPI_ISL_4877431
EPI_ISL_4877432
EPI_ISL_4877434
EPI_ISL_4877800
EPI_ISL_4877848
EPI_ISL_4877852
EPI_ISL_4877856
EPI_ISL_4877861
EPI_ISL_4877881
EPI_ISL_4877938
EPI_ISL_4877961
EPI_ISL_4878084
EPI_ISL_4878085
EPI_ISL_4878167
EPI_ISL_4878169
EPI_ISL_4878170
EPI_ISL_4878172
EPI_ISL_4878179
EPI_ISL_4878190
EPI_ISL_4878204
EPI_ISL_5530707
EPI_ISL_5530695
EPI_ISL_5530708
EPI_ISL_5530696
EPI_ISL_5531677
EPI_ISL_5531783
EPI_ISL_5530692
EPI_ISL_5530899
EPI_ISL_5531662
EPI_ISL_5530697
EPI_ISL_5530914
EPI_ISL_5531665
EPI_ISL_5530710
EPI_ISL_5531372
EPI_ISL_5530701
EPI_ISL_5530905
EPI_ISL_5531357
EPI_ISL_5530906
EPI_ISL_5531595
EPI_ISL_5531596
EPI_ISL_5530907
EPI_ISL_5531594
EPI_ISL_5530712
EPI_ISL_5531632
EPI_ISL_5531597
EPI_ISL_5531523
EPI_ISL_5531602
EPI_ISL_5531601
EPI_ISL_1378670
EPI_ISL_5531545
EPI_ISL_5531600
EPI_ISL_1404589
EPI_ISL_1364433
EPI_ISL_1469541
EPI_ISL_1469542
EPI_ISL_1469540
EPI_ISL_1378672
EPI_ISL_1378671
EPI_ISL_1501676
EPI_ISL_1378644
EPI_ISL_1378645
EPI_ISL_1399162
EPI_ISL_1399163
EPI_ISL_5531644
EPI_ISL_1404598

EPI_ISL_1404599
EPI_ISL_1404600
EPI_ISL_1423007
EPI_ISL_1423006
EPI_ISL_1422992
EPI_ISL_5531544
EPI_ISL_5531546
EPI_ISL_1422993
EPI_ISL_1422994
EPI_ISL_1422995
EPI_ISL_1422996
EPI_ISL_1422997
EPI_ISL_1422998
EPI_ISL_1422999
EPI_ISL_1423000
EPI_ISL_1469493
EPI_ISL_1469545
EPI_ISL_1469547
EPI_ISL_1469546
EPI_ISL_1469544
EPI_ISL_1469543
EPI_ISL_1469495
EPI_ISL_5531603
EPI_ISL_1469496
EPI_ISL_1627471
EPI_ISL_1501683
EPI_ISL_1483253
EPI_ISL_1483254
EPI_ISL_1483255
EPI_ISL_1501675
EPI_ISL_5531549
EPI_ISL_5531605
EPI_ISL_1501677
EPI_ISL_1501678
EPI_ISL_5530682
EPI_ISL_5531604
EPI_ISL_1511631
EPI_ISL_4877438
EPI_ISL_1511632
EPI_ISL_1511633
EPI_ISL_5530702
EPI_ISL_1575294
EPI_ISL_1575292
EPI_ISL_1575291
EPI_ISL_1575293
EPI_ISL_1575253
EPI_ISL_1575256
EPI_ISL_1575257
EPI_ISL_4877886
EPI_ISL_1575258
EPI_ISL_5531606
EPI_ISL_1575283
EPI_ISL_1575262
EPI_ISL_1575282
EPI_ISL_1575259
EPI_ISL_1575260
EPI_ISL_1585920
EPI_ISL_1620636
EPI_ISL_1620637
EPI_ISL_2992004
EPI_ISL_1675159
EPI_ISL_1663881
EPI_ISL_5531683
EPI_ISL_5531609
EPI_ISL_1675161
EPI_ISL_1675160
EPI_ISL_5531540
EPI_ISL_5531588
EPI_ISL_5531791
EPI_ISL_1675162
EPI_ISL_5531610
EPI_ISL_1675152
EPI_ISL_1675153
EPI_ISL_5531518
EPI_ISL_1675154
EPI_ISL_5531817
EPI_ISL_2992042
EPI_ISL_5531611
EPI_ISL_5531664
EPI_ISL_5531551
EPI_ISL_5531666
EPI_ISL_5531589
EPI_ISL_5531787
EPI_ISL_2211125
EPI_ISL_5531647
EPI_ISL_5531633
EPI_ISL_5531492
EPI_ISL_5531634
EPI_ISL_5531653
EPI_ISL_5531615
EPI_ISL_5531614
EPI_ISL_4877796
EPI_ISL_2566121
EPI_ISL_2566122
EPI_ISL_5530719
EPI_ISL_5531966
EPI_ISL_2992019
EPI_ISL_4877698
EPI_ISL_4877699
EPI_ISL_5531636
EPI_ISL_5531635
EPI_ISL_4878081
EPI_ISL_5531557
EPI_ISL_4878109
EPI_ISL_2612503
EPI_ISL_5531538
EPI_ISL_4877964
EPI_ISL_5531638
EPI_ISL_2612501
EPI_ISL_2612504
EPI_ISL_4877891
EPI_ISL_4877892
EPI_ISL_4877893
EPI_ISL_4877894
EPI_ISL_4877895
EPI_ISL_4877899
EPI_ISL_5530725
EPI_ISL_5530939
EPI_ISL_5530724
EPI_ISL_5531381
EPI_ISL_5530723
EPI_ISL_5531094
EPI_ISL_5531961
EPI_ISL_5531668
EPI_ISL_2690274
EPI_ISL_2690277
EPI_ISL_5531102
EPI_ISL_2690278
EPI_ISL_2690279
EPI_ISL_2690273
EPI_ISL_5530726
EPI_ISL_2690275
EPI_ISL_5531495
EPI_ISL_4877896
EPI_ISL_5531617
EPI_ISL_2690282
EPI_ISL_2690283
EPI_ISL_4877897
EPI_ISL_4878121
EPI_ISL_4877445
EPI_ISL_5530729
EPI_ISL_5530728
EPI_ISL_5530731
EPI_ISL_5530736
EPI_ISL_5530969
EPI_ISL_5531618
EPI_ISL_5530737
EPI_ISL_5531642
EPI_ISL_5531643
EPI_ISL_5530705
EPI_ISL_2801516
EPI_ISL_2801479
EPI_ISL_2801518
EPI_ISL_5531470
EPI_ISL_2801519
EPI_ISL_2801520
EPI_ISL_2801495
EPI_ISL_2801508
EPI_ISL_5531107
EPI_ISL_5530738
EPI_ISL_2801521
EPI_ISL_2801483

Table S2: Ct values and SARS-CoV-2 concentrations in the collected samples

Sample Date	Sample Location	Ct		Average Concentration (gc/L)	
		N1	N2	N1	N2
November 2020	(1) Pooled, samples from three facilities	30.31-35.67	30.25-35.75	163416	163097
1/1/21	(2) TMWRF Sewershed	35.3	33.19	72505	143398
1/10/21	(3) TMWRF-Reno interceptor	36.57	36.35	38457	53370
1/29/21	(4) STMWRF sewershed	36.93	40.97	67001	20394
3/11/21	TMWRF Sewershed	35.57	35.03	139150	257841
3/18/21	TMWRF Sewershed	36.75	36.88	54283	44184
6/20/21	TMWRF Sewershed		36.15		30799

Note: (1) Pooled samples: Total RNA were extracted from the wastewater sampels that collected from TMWRF, STMWRF, and RSWRF, and then mixed together for sequencing

(2) TMWRF: Truckee Meadows Water Reclamation Facility

(3) TMWRF-Reno interceptor: Wastewater were collected from Reno sewer network, and then received in TMWRF

(4) STMWRF: South Truckee Meadows Water Reclamation Facility

Table S3: Sequencing informaiton (samples collected in June 2021)

Chromosome	Region	Type	Reference	Alleles (Count/Coverage)	Length	Non-synonymous	Gene	1st predominant signature (% Freqeuency)	Associated variants (1st)	2nd predominant signature (% Frequency)	Associated variants (2nd)	3rd signature (% Frequency)	Associated variants (3rd)	Signatures in Variants Being Monitored	Note
MN908947.3	1104	SNV	T	T (8/15), C (7/15)	1	Yes	ORF1a	1104T: I280-wt (53%)	all others	1104T>C: I280T (47%)	some 21A (Delta)				Not an unique signature
MN908947.3	3267	SNV	C	T (9/16), C (7/16)	1	Yes	ORF1a	3267C>T: T1001I (56%)	20I (Alpha)	3267C: T1001-wt (44%)	all other variants			B.1.1.7	
MN908947.3	5284	SNV	C	T (10/19), C (9/19)	1	No	ORF1a	5284C>T (53%)	20I (Alpha)	5284C-wt (47%)	all other variants				Not an unique signature
MN908947.3	5388	SNV	C	A (16/19), C (3/19)	1	Yes	ORF1a	5388C>A: A1708D (84%)	20I (Alpha)	5388C: A1708-wt (16%)	all other variants			B.1.1.7	
MN908947.3	18877	SNV	C	T (623/645), C (22/645)	1	No	ORF1a	18877C>T (97%)	21H	18877C-wt (3%)	all other variants				Not an unique signature
MN908947.3	21987	SNV	G	A (554/613), G (40/613), T (19/613)	1	Yes	S	21987G>A: G142D (90%)	Primarily 21A (Delta) and some 20I (Alpha)	21987G: G142-wt (7%)	all other variants	21987G>T: G142V (3%)	Some 20I (Alpha)		Not an unique signature
MN908947.3	22029..22034	Deletion	AGTTCA	Deletion-: (785/838)	6	Yes	S	22029..22034: E156_R158delinsG (93%)	21A (Delta)	22029..22034-wt (7%)	all other variants			B.1.617.2	
MN908947.3	23012	SNV	G	A (585/593), G (8/593)	1	Yes	S	23012G>A: E484K (98%)	Beta, Gamma, Kappa and 20B	23012G: E484-wt (2%)	all other variants			B.1.1.7, B.1.351, P.1, B.1.526	
MN908947.3	23403	SNV	A	G (42/44), A (2/44)	1	Yes	S	23403A>G: D614G (95%)	almost all variants	23403A: D614-wt (5%)	19A/19B and some Alpha				Not an unique signature
MN908947.3	23416	SNV	A	T (38/63), A (25/63)	1	No	S	23416A>T (60%)	20E and 20I (Alpha)	23416A-wt (40%)	all other variants				Not an unique signature
MN908947.3	25352	SNV	G	T (27/39), G (12/39)	1	Yes	S	25352G>T: V1264L (69%)	some 21A (Delta)	25352G: V1264-wt (31%)	all other variants				Not an unique signature
MN908947.3	25469	SNV	C	T (43/50), C 7/50)	1	Yes	ORF3a	25469C>T: S26L (86%)	21A (Delta)	25469C: S26-wt (14%)	all other variants			B.1.617.2	
MN908947.3	26767	SNV	T	C (364/377), T 13/377)	1	Yes	M	26767T>C: I82T (97%)	21A (Delta), Epsilon, 20F	26767T: I82-wt (3%)	all other variants			B.1.617.2	
MN908947.3	27972	SNV	C	C 11/17), T (6/17)	1	Yes	ORF8	27972C: Q27 (65%)	all others	27972C>T: Q27* (35%)	20I (Alpha)			B.1.1.7	
MN908947.3	28271	Deletion	A	Deletion-: (7/11), A (3/11), G (1/11)	1	-		28271A>Deletion-: 63%	21A (Delta) and 20I (Alpha)	28271A-wt (27%)	all other variants	28271A>G (9%)	20F	B.1.617.2, B.1.617.1	
MN908947.3	28881..28883	MNV	GGG	AAC (8/10), GGG (2/10)	3	Yes	N	28881-28883GGG>AAC: R203_G204>203K_204R (80%)	20B, 20D, 20E (Theta), 20F, 20G (Lambda), 20I (Alpha), 20J (Gamma)	28881-28883GGG: R203_G204 (20%)	all other variants				Not an unique signature
MN908947.3	28977	SNV	C	T (8/12), C (4/12)	1	Yes	N	28977C>T: S235F (66%)	20I (Alpha)	28977C: S235 (33%)	all other variants			B.1.1.7	
MN908947.3	29735	SNV	A	C (5811/5850), T (20/5850), A (14/5850)	1	-		29735A>C (99%)	Not found	29735A>T (0.3%)	Not found	29735A-wt (0.2%)	All variants		Deleted in some 21B
MN908947.3	29758	SNV	T	G (8296/8328), T (22/8328)	1			29758T>G (99%)	None	29758T-wt (0.25%)	All variants				Deleted in some 21B

Table S4: Sequencing informaiton (Samples collected in March 2021)

Chromosome	Region	Type	Reference	Alleles (Count/Coverage)	Length	Non-synonymous	Gene	1st predominant signature (% Freqeuency)	Associated variants (1st)	2nd predominant signature (% Frequency)	Associated variants (2nd)	3rd signature (% Frequency)	Associated variants (3rd)	Signatures in Variants Being Monitored	Note
MN908947.3	3078	SNV	A	C (69/69)	1	Yes	ORF1a	3078A>C: E938A (100%)	Not Reported						E938K is in some 20A
MN908947.3	3467	SNV	G	T (23/23)	1	Yes	ORF1a	3467G>T: G1068* (100%)	Not Reported						G1068V is in some 20J
MN908947.3	3939	SNV	G	T (10/10)	1	Yes	ORF1a	3939G>T: R1225I	Some 20I (Alpha)						Not an unique signature
MN908947.3	4815	SNV	C	T (15/15)	1	Yes	ORF1a	4815C>T: S1517F (100%)	Some 20A						
MN908947.3	8516	SNV	G	A (32/32)	1	Yes	ORF1a	8516G>A:V2751I (100%)	Not Reported						V2751F is in some 20I (Alpha)
MN908947.3	9072	SNV	C	T (48/48)	1	Yes	ORF1a	9072C>T: T2936I (100%)	20B						Present in B.1.1.318 lineage
MN908947.3	9891	SNV	C	T (32/32)	1	Yes	ORF1a	9891C>T: A3209V (100%)	21A (Delta), 20B and 20C					B.1.617.2, B.1	Present in B.1.1.318 and B.1.526
MN908947.3	10116	SNV	C	T (24/25), C (1	1	Yes	ORF1a	10116C>T: T3284I (96%)	20B	10116C: T3284 (4%)				B.1.1.318	Present in B.1.1.318 lineage
MN908947.3	11478	SNV	T	A (36/69), T 3	1	Yes	ORF1a	11478A>T: I3738N 52%)	Not Reported	11478A: I373	All variants				
MN908947.3	14228	SNV	A	T (43/43)	1	Yes	ORF1b	14228A>T: L254M (100%)	Not Reported						
MN908947.3	14408	SNV	C	T (47/47)	1	Yes	ORF1b	14408C>T: P314L (100%)	All variants 20A onwards						
**MN908947.3**	**21646**	**SNV**	**C**	**T (37/37)**	**1**	**No**	**S**	**21646 C>T (100%)**	**Some 20I (Alpha)**						
**MN908947.3**	**23012**	**SNV**	**G**	**A (36/36)**	**1**	**Yes**	**S**	**23012G>A:E484K (100%)**	**20H (Beta), 20J (Gamma), 21D (Eta), 21F (Iota)**					**P.1, B.1.351**	
**MN908947.3**	**23403**	**SNV**	**A**	**G (30/30)**	**1**	**Yes**	**S**	**23403A>G: D614G (100%)**	**almost all variants**						
**MN908947.3**	**23604**	**SNV**	**C**	**A (40/40)**	**1**	**Yes**	**S**	**23604C>A: P681H (100%)**	**20A, 20B, 20I (Alpha)**					**B.1.1.7**	
**MN908947.3**	**24382**	**SNV**	**C**	**T (15/15)**	**1**	**No**	**S**	**24382C>T (100%)**	**20B**					**B.1.1.318**	**Present in B.1.1.318 lineage**
MN908947.3	26767	SNV	T	C (56/56)	1	Yes	M	26767T>C: L82T (100%)	21A (Delta), 21D (Eta)					B.1.617.2	
MN908947.3	27143	SNV	C	T (40/40)	1	No	M	27143C>T (100%)	some 20H and 20B						
MN908947.3	27996	SNV	G	T (30/30)	1	Yes	ORF8	27996G>T: D35Y (100%)	21D (Eta)						
MN908947.3	28209	SNV	G	T (70/71)	1	Yes	ORF8	28209G>T: E106* (100%)	20I (Alpha)					B.1.1.7	
MN908947.3	28271	SNV	A	G (45/45)	1	-		28271A>G (100%)	Some 20I (Alpha)					B.1.1.7	
MN908947.3	28881..2888	MNV	GGG	AAC 60/60)	3	Yes	N	28881-28883GGG>AAC: R203_	20B, 20D, 20E (Theta), 20F, 20G (Lambda), 20I (Alpha), 20J (Gamma)					B.1.1.7	
MN908947.3	28896..2889	Deletion	CTA	Deletion (60/	3	Yes	N	28881..28883: A208_R209del	20B					B.1.1.318	Present in B.1.1.318 lineage
MN908947.3	29769	SNV	C	T (64/64)	1	-		29769C>T (100%)	20B					B.1.1.318	Present in B.1.1.318 lineage

Table S5: Sequencing informaiton (Samples collected in January 2021)

Chromosome	Region	Type	Reference	Alleles (Count/Coverage)	Length	Non-synonymous	Gene	1st predominant signature (% Freqeuency)	Associated variants (1st)	2nd predominant signature (% Frequency)	Associated variants (2nd)	3rd signature (% Frequency)	Associated variants (3rd)	Signatures in Variants Being Monitored	Note
MN908947.3	241	SNV	C	T (331/331)	1	-		241 C>T (100%)	All 20A onwards						Not an unique signature
MN908947.3	835	SNV	C	T (3982/4305), C (309/4305)	1	No	ORF1a	835C>T: F190-wt (93%)	some 20B, 20G and 21D	835C: F190-wt (7%)	All variants				Not an unique signature
MN908947.3	1059	SNV	C	T (3742/3745), A (3/3745)	1	Yes	ORF1a	1059C>T: T265I (99.9%)	20A, 20C, 20G, 21C, 21F and 20H	1059C>A: T265N (0.07%)	Not reported			B.1.427/429 (Epsilon), 21F (Iota)	
MN908947.3	2647	SNV	A	G (600/1437), A (836/1437)	1	No	ORF1a	2647A: K794-wt (58%)	All variants	2647A>G: K794 (42%)	20D				Not an unique signature
MN908947.3	2649	SNV	A	G (598/1432), A (831/1432), T (3/1432)	1	Yes	ORF1a	2649A: D795-wt (58%)	All variants	2649A>G: D795G (41.8%)	some 20I (Alpha)	2649A>T: D795V (0.2%)	Not Reported		D795N present in some 21A (Delta)
MN908947.3	3037	SNV	C	T (21/22), C 91/22)	1	No	ORF1a	3037C>T (95%)	All 20A onwards	3037C-wt (5%)	19A and 19B				
MN908947.3	9426	SNV	C	T (1040/1328), C (288/1328)	1	Yes	ORF1a	9426C>T: A3054V (78%)	Not Reported	9426C: A3054-wt (22%)	all variants				
MN908947.3	12100	SNV	C	T (215/297), C (82/297)	1	No	ORF1a	12100C>T (72%)	21C (Epsilon)	12100C-wt (28%)	all other variants			B.1.427/429 (Epsilon)	
MN908947.3	12878	SNV	A	G (713/777), A (64/777)	1	Yes	ORF1a	12787A>G: I4205V (91.6%)	21C (Epsilon)	12787A: I4205-wt (8.4%)	all other variants			B.1.427/429 (Epsilon)	
MN908947.3	13261	SNV	C	T (3778/5273), C (1478/5273), A (17/5273)	1	No	ORF1a	13261C>T (71.6%)	some 20G, 21A and 20I	13261C-wt (28%)	All variants	13261C>A: C4332*(0.3%)			Not an unique signature
MN908947.3	14408	SNV	C	T (1022/1026), C (2/1026), A (2/1026)	1	Yes	ORF1b	14408C>T: P314L (99.6%)	All variants 20A onwards	14408C>A: P314H (0.2%)	some 20H variants	14408C: P314-wt (0.2%)	19A and 19B		Not an unique signature
MN908947.3	19390	SNV	C	T (201/257), A (57/257)	1	Yes	ORF1b	19390C>T: P1975S (78%)	some 20I (Alpha)	19390C: P1975-wt (22%)	All variants				Not an unique signature
**MN908947.3**	**22917**	**SNV**	**T**	**G (178/252), T (73/252), A (1/252)**	**1**	**Yes**	**S**	**22917T>G:L452R (70.6%)**	**Cal.20C, 21A (Delta), 21B (Kappa)**	**22917T:L452-wt (29%)**	**all other variants**	**22917T>A:L452Q (0.4%)**	**21G (Lambda)**	**B.1.526 (Iota), B.1.427, B.1.429, B.1.617.1 (Kappa), B.1.617.3, and the lineages and sub-lineages designated Delta (B.1.617.2, AY.1, AY.2, and AY.3)**	
**MN908947.3**	**23403**	**SNV**	**A**	**G (1385/1387), A (2/1387)**	**1**	**Yes**	**S**	**23403A>G: D614G (99.7%)**	**almost all variants**	**23403A: D614-wt (0.14%)**					**Not an unique signature**
**MN908947.3**	**24134**	**SNV**	**C**	**T (387/412), C (25/412)**	**1**	**Yes**	**S**	**24134C>T: L858F (94%)**	**some 20B**	**24134C: L858-wt (6%)**	**All variants**				**Not an unique signature**
MN908947.3	25563	SNV	G	T (128/128)	1	Yes	ORF3a	25563G>T: Q57H (100%)	20C, 20G, 20H, 21C and 21F					B.1.526 (Iota), B.1.427, B.1.429,	
MN908947.3	25907	SNV	G	T (6822/6857), A (28/6857), G (7/6857)	1	Yes	ORF3a	25907G>T: G172V (99.5%)	20G	25907G>A: G172D (0.4%)	19A	25907G: G172-wt (0.1%)	All variants		
MN908947.3	27964	SNV	C	T (6916/7681), C (765/7681)	1	Yes	ORF8	27964C>T: S24L (90%)	20G	27964C: S24-wt (10%)	all other variants				
MN908947.3	28472	SNV	C	T (2713/3793), C (1079/3793)	1	Yes	N	28472C>T: P67S (71.4%)	20G	28472C: P67-wt (28.4%)	all other variants				
MN908947.3	28869	SNV	C	T (1015/1332), C (315/1332), A (2/1332)	1	Yes	N	28869C>T: P199L (76.2%)	21F and 21C (Epsilon)	28869C: P199-wt (23.6%)	all other variants	28869C>A: P199Q (0.15%)	20B		
MN908947.3	29362	SNV	C	T (123/126), C (3/126)	1	No	N	29362C>T (97.6%)	21C (Epsilon)	29362C-wt (2.4%)	all other variants				

Table S6: Sequencing informaiton (Samples collected in November 2020)

Chromosome	Region	Type	Reference	Alleles (Count/Coverage)	Length	Non-synonymous	Gene	1st predominant signature (% Freqeuency)	Associated variants (1st)
MN908947.3	10560	SNV	C	T (3738/4659), C 912/4659), A (9/4659)	1	Yes	ORF1a	10560C>T: T3432I (80%)	Not Reported
MN908947.3	10563	SNV	G	T (3744/4611), G (863/4611), A 3/4611)	1	Yes	ORF1a	10563G>T: G3433V (81%)	Not Reported
MN908947.3	12513	SNV	C	T (14/14)	1	Yes	ORF1a	12513C>T: T4083M (100%)	many variants, 20C, 20G and 20I
MN908947.3	14333	SNV	G	A (3083/3691), G (606/3691)	1	Yes	ORF1b	14333G>A: C289T (83.5%)	Not Reported
MN908947.3	14408	SNV	C	T (15901/15955), A (34/15955), C (19/15955)	1	Yes	ORF1b	14408C>T: P314L (99.6%)	All variants 20A onwards
MN908947.3	21595	SNV	C	T (2660/2663), C (3/2663)	1	No	S	21595C>T (99.9%)	some variants of 20C, 20G and 20I
MN908947.3	21707	SNV	C	T (1890/1892), A (2/1890)	1	Yes	S	21707C>T: H49Y (99.9%)	some variants of 20B, 20G and 20I
MN908947.3	23403	SNV	A	G 61498/61653), A (119/61653)	1	Yes	S	23403A>G: D614G (99.7%)	almost all variants
MN908947.3	23426	SNV	G	T (63326/63816), G (415/63816), A (45/63816)	1	Yes	S	23426G>T: V622F (99.2%)	some 19B and 20D, 20I
MN908947.3	28019	SNV	T	C (86/111), T (25/111)	1	No	ORF8	28019T>C (77%)	Some 20B

Chromosome	Region	Type	Reference	2nd predominant signature (% Frequency)	Associated variants (2nd)	3rd signature (% Frequency)	Associated variants (3rd)	Signatures in Variants Being Monitored	Note
MN908947.3	10560	SNV	C	10560C: T3432-wt (19%)	All variants	10560C>A: T3432N (0.2%)	Not Reported		T3432* in few 21A (Delta)
MN908947.3	10563	SNV	G	10563G: G3433-wt (18%)	All variants	10563G>A: G3433E (0.07%)	Not Reported		T3433* in few 21A (Delta)
MN908947.3	12513	SNV	C						
MN908947.3	14333	SNV	G	14333G: C289-wt (16.4%)	All variants				
MN908947.3	14408	SNV	C	14408C>A: P314H (0.2%)	some 20H variants	14408C: P314-wt (0.12%)	19A and 19B		Not an unique signature
MN908947.3	21595	SNV	C	21595C-wt (0.1%)	All variants			B.1.1.7	Not an unique signature
MN908947.3	21707	SNV	C	21707C>A: H49N (0.1%)	Not Reported			B.1.1.7	Not an unique signature
MN908947.3	23403	SNV	A	23403A: D614-wt (0.2%)	19A and 19B				Not an unique signature
MN908947.3	23426	SNV	G	23426G: V622-wt (0.6%)	All variants	23426G>A: V622I (0.1%)	some 20C	B.1.1.7	Not an unique signature
MN908947.3	28019	SNV	T	28019T-wt (23%)	All variants				Not an unique signature
